# AT1R-Mediated Apoptosis of Bone Marrow Mesenchymal Stem Cells Is Associated with mtROS Production and mtDNA Reduction

**DOI:** 10.1155/2019/4608165

**Published:** 2019-10-21

**Authors:** Fenxi Zhang, Zimei Dong, Shuai Gao, Guangwen Chen, Dezeng Liu

**Affiliations:** College of Life Science, Henan Normal University, Xinxiang, 453007 Henan, China

## Abstract

Angiotensin II (Ang II) is used as an inducer for the differentiation of mesenchymal stem cells (MSCs). Whether the commonly used doses of Ang II for MSC differentiation affect cell apoptosis has not been elucidated. In this study, we investigated the effect of Ang II on the apoptosis of bone marrow MSCs (BMMSCs), and its relations to the activation of Ang II receptor-1- (AT1R-) signaling, mitochondrial ROS (mtROS) generation, and mitochondrial DNA (mtDNA) leakage. AT1R expression in BMMSCs was identified by immunostaining and Western-blotting assays. BMMSC viability was measured by MTT assay following exposure to 1 nM~1 mM Ang II for 12 hours. Cell apoptosis, mtROS, and mtDNA levels were detected by FAM-FLICA® Poly Caspase, MitoSOX™ superoxide, and PicoGreen staining, respectively. The expressions of Bcl2 and Bax were measured by Western-blotting assays. Next, we used losartan to block AT1R-signaling and subsequently measured apoptosis, mtROS, and mtDNA levels, again. The maximum viability of BMMSCs was in response to 100 nM Ang II, after that it began to decrease with the increase of Ang II doses, indicating that Ang II (≧1 *μ*M) may cause apoptosis of BMMSCs. As expected, 1 *μ*M and 10 *μ*M Ang II both caused BMMSC apoptosis. Furthermore, 1 *μ*M and 10 *μ*M Ang II could also induce mtROS generation and cause a marked mtDNA leakage. The application of losartan markedly inhibited Ang II-induced mtROS production, mtDNA leakage, and BMMSC apoptosis. In conclusion, the activation of AT1R-signaling stimulates apoptosis of BMMSCs, which is associated mtROS production and mtDNA reduction.

## 1. Introduction

Mesenchymal stem cells (MSCs) are a kind of multipotent cells that can differentiate into a series of cell lineages, including osteocytes, adipocytes, chondrocytes, endothelial cells, cardiomyocytes, and neurons when exposed to appropriate conditions [1, 2]. Bone marrow MSCs (BMMSCs) are the most widely used stem cells in tissue engineering. It has been proposed that transplantation of BMMSCs ameliorates symptoms of many diseases such as Alzheimer's disease, heart infarction, stroke, rheumatoid arthritis, and pulmonary injury [3–8].

Angiotensin II (Ang II) is a peptide hormone that mediates most functions of the renin-angiotensin system (RAS), and participates in many physiological and pathophysiological processes including angiogenesis, cell proliferation, differentiation, apoptosis, and inflammation in vascular endothelial cells, cardiomyocytes, and smooth muscle cells [9–11]. Ang II exerts its effects mainly through activating its receptor type-1 (AT1R) [11]. Ang II has been widely used as an inducer to stimulate the differentiation of MSCs to functional cells such as cardiomyocytes, endothelial cells, adipocytes, and smooth muscle-like cells [12–16]. The doses of Ang II usually used to stimulate differentiation of MSCs were from 0.1 *μ*M to 10 *μ*M [12–14].

Several lines of evidence suggest that an appropriate amount of Ang II has the potential to stimulate proliferation of many cell lineages including endothelial cells, cardiac fibroblasts, vascular smooth muscle cells, and hematopoietic progenitor cells [17–20]. However, high concentrations of Ang II had been reported to cause the apoptosis of endothelial cells, smooth muscle cells, and kidney tubular cells [21–23]. Recent studies had also shown that Ang II could induce the production of mitochondrial reactive oxygen species (mtROS) in the pathogenesis of cardiovascular diseases [24, 25]. Kim et al. observed that the apoptosis of kidney tubular cells induced by Ang II was mediated by the activation of mitochondrial NADPH oxidase 4 (NOX4) and the increase of ROS production [23]. We hypothesized that the commonly used doses of Ang II may lead to the apoptosis of BMMSCs, which may be related to AT1R-mediated mtROS production and mitochondrial DNA (mtDNA) reduction.

## 2. Materials and Methods

### 2.1. Materials

Ang II, losartan, and dihydroethidium (DHE) were purchased from Sigma-Aldrich (Shanghai, China). The MitoSOX™ superoxide indicator and the PicoGreen kit were obtained from Invitrogen (Carlsbad, CA, USA). The AT1R primary antibody and secondary antibodies were obtained from Abcam (Cambridge, MA, USA), and Bax, Bcl2, and *β*-actin antibodies were from Santa Cruz Biotechnology Inc. (Santa Cruz, CA, USA). The ECL Western-blotting substrate was purchased from Thermo Fisher Scientific (Rockford, IL, USA). The FAM-FLICA® Poly Caspase Assay Kit was purchased from ImmunoChemistry Technologies LLC (Bloomington, MN, USA). The MTT assay kit was from ATCC (Manassas, VA, USA).

### 2.2. Isolation and Culture of BMMSCs

BMMSCs were isolated and cultured according to recently published protocols [26–28]. In brief, BMMSCs were isolated from bone marrow that was harvested from mouse tibias and femurs, then plated into 100 mm petri dishes and cultured in DMEM supplemented with 15% FBS, 2 mM L-glutamine, 100 U/mL penicillin, and 100 g/mL streptomycin for 3 hours. Then, the nonadherent cells were removed and the medium was replaced with a fresh one. A purified population of BMMSCs was obtained after 3 weeks of culture. BMMSCs were from 8 mice, and the 3rd passage BMMSCs were used in the experiments.

### 2.3. Cell Viability Assay

Cell viability was measured by MTT assay according to recently published protocols [27, 28]. In brief, BMMSCs (1 × 10^3^) were plated into 96-well plates. After BMMSCs attached to the plates, they were exposed to different concentrations of Ang II (1 nM~1 mM) for 12 hours. Then, 10 *μ*L MTT reagent was added into each well and the plates were incubated for 3 hours at 37°C. Then, 100 *μ*L detergent reagent was added to each well and the plates were incubated at room temperature in the dark for an additional 2 hours. Absorbance at 570 nm was measured with a microplate reader.

### 2.4. Immunofluorescence Assay

Immunofluorescent staining was performed using the standard protocols [27, 28]. In brief, the third passage BMMSCs grown on the round coverslips were fixed with 4% buffered paraformaldehyde at room temperature for 15 min. The cells on the coverslips were treated with 0.1% Triton X-100 at room temperature for 3 min and then blocked with 1% BSA. The cells were incubated with rabbit anti-mouse AT1R antibody (1 : 400) at 4°C for 1 hour and then washed with PBS for 3 times. After washing, the cells were incubated with FITC-conjugated duck anti-rabbit secondary antibody (1 : 1000) in the dark. After washing, the coverslips were mounted on slides with the Prolong Gold Antifade Reagent and imaged with a fluorescence microscope.

### 2.5. Western-Blotting Assay

Proteins were extracted from BMMSCs and separated by 10% SDS-PAGE. After electrophoresis, proteins were transferred onto the PVDF membranes. The membranes were blocked with 5% BSA or 5% nonfat milk (as the manufacturer's instructions) in TBS-T and then incubated with AT1R, Bax, Bcl2, or *β*-actin antibodies (1 : 2000) at 4°C overnight. Then, the blots were incubated with HRP-conjugated secondary antibodies (1 : 10000) for 1 hour at room temperature. The immunoreactive bands were detected by enhanced chemiluminescence.

### 2.6. Cell Apoptotic Assay

Cell apoptosis was detected by Poly Caspase and DAPI staining with a FAM-FLICA® Poly Caspase Assay Kit following the kit's instructions and recently published protocols [28]. Briefly, the 3rd passage BMMSCs were seeded in a 24-well plate with 10 mm round coverslips (pretreated with polylysine). The cells were then exposed to the FLICA reagent and incubated at 37°C for 1 hour in a wet box. The cells were then treated with DAPI for 10 min at room temperature. The cells were washed with PBS, mounted on slides with the ProLong Gold Antifade Reagent and viewed under a fluorescence microscope. The apoptotic rates were calculated by counting the numbers of caspase-fluorescence-positive cells and the cells with condensed and/or fragmented nuclei (DAPI staining).

### 2.7. Mitochondrial ROS Measurement

BMMSCs treated with different concentrations of Ang II (or pretreated with the AT1R blocker losartan) were stained with 10 *μ*M of the MitoSOX™ superoxide indicator according to the manufacturer's instructions and imaged with a fluorescence microscope.

### 2.8. Mitochondrial DNA Staining

BMMSCs treated with different concentrations of Ang II (or pretreated with losartan) were incubated with 5 *μ*M PicoGreen for 1 hour at 37°C, washed with PBS, and imaged with a fluorescence microscope.

### 2.9. Statistical Analysis

Statistical analysis was performed with the SPSS15.0 software. Data were presented as the means ± standard deviations (SDs). Univariate comparisons of means were evaluated using Student's *t*-tests for comparison between two groups or one-way ANOVA with Tukey's post hoc adjustment for multiple comparisons when appropriate. *P* < 0.05 was considered a statistically significant difference.

## 3. Results

### 3.1. Identification of AT1R Expression in BMMSCs

**Figure 1:**
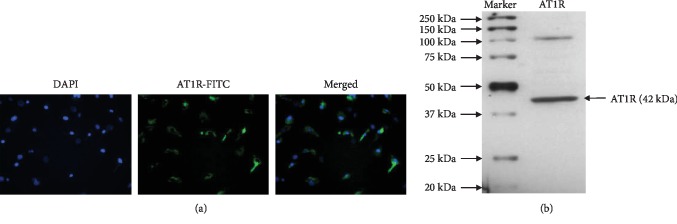
Identification of AT1R expression in BMMSCs. (a) Immunostaining assay showing AT1R expression. (b) Western-blotting assay showing AT1R protein expression in BMMSCs.

AT1R is the most important receptor for Ang II, and its activation has been proven to promote cell differentiation and proliferation. Although Ang II has been widely utilized to stimulate the differentiation of MSCs, the status of the AT1R expression in BMMSCs has not been elucidated. In this study, the AT1R expression in BMMSCs was identified by immunostaining and Western-blotting assays. As shown in Figure 1, the immunostaining assay showed a positive expression of AT1R in BMMSCs (Figure 1(a)), which was further confirmed by the Western-blotting (Figure 1(b)) assay.

### 3.2. Effect of Ang II on the Proliferation of BMMSCs

**Figure 2:**
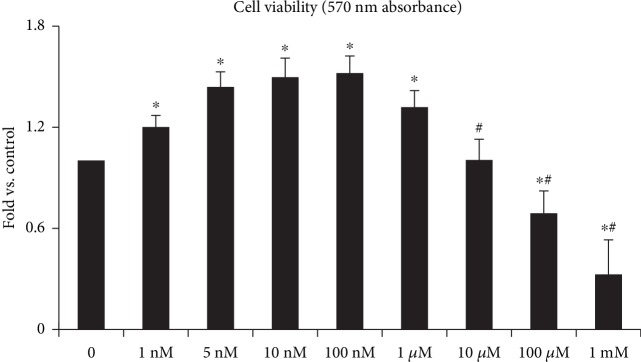
MTT assay showing cell viability of BMMSCs after exposure to different concentrations of Ang II (1 nM~1 mM) for 12 hours. Bar graphs represent mean ± SD (*n* = 5 per group). ^∗^*P* < 0.05 vs. control (0 nM) and ^#^*P* < 0.05 vs. the 10 nM Ang II group.

As shown in Figure 2, low concentrations of Ang II (1 nM~100 nM) could increase the viability of BMMSCs (*P* < 0.01), but high concentrations of Ang II (10 *μ*M~1 mM) markedly inhibited BMMSC proliferation. The plateau of BMMSC proliferation in response to Ang II was 100 nM; after that, cell viability began to decrease (Figure 2). This data suggests that Ang II (≧1 *μ*M) may cause the apoptosis of BMMSCs. Based on cell viability data, the doses of 100 nM, 1 *μ*M, and 10 *μ*M were selected to study the effects of Ang II on apoptosis, mtROS production, and the integrity of mtDNA in BMMSCs.

### 3.3. Effect of Ang II on the Apoptosis of BMMSCs

**Figure 3:**
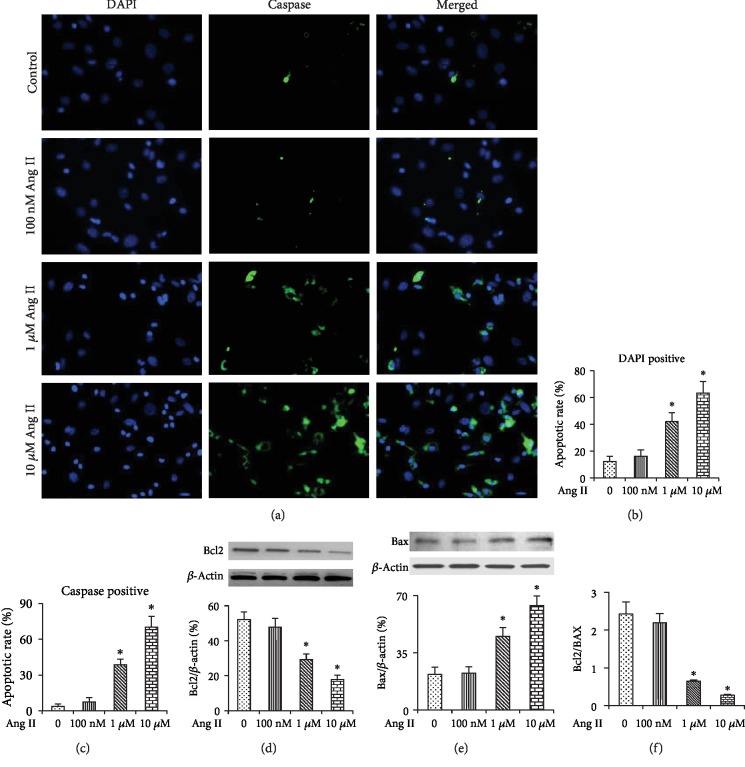
Apoptosis of BMMSCs after treatment with 0, 100 nM, 1 *μ*M, and 10 *μ*M Ang II for 12 hours. (a) Poly Caspase and DAPI staining showing apoptosis of BMMSCs. (b) Quantification of the numbers of caspase-positive cells. (c) Quantification of the numbers of the cells with condensed and/or fragmented nuclei. (d and e) Western-blotting assay showing Bcl2 and Bax expressions in BMMSCs after exposure to different doses of Ang II. (f) The ratio of Bcl2/Bax. Bar graphs represent mean ± SD (*n* = 4 per group). ^∗^*P* < 0.05 vs. control.

Cell apoptosis was detected by FAM-FLICA® Poly Caspase and DPAI staining assays. As shown in Figure 3, Poly Caspase staining and DAPI staining both showed that 1 *μ*M and 10 *μ*M Ang II significantly increased apoptosis as compared with the control (0 nM) group (*P* < 0.01); however, 100 nM Ang II did not significantly affect the apoptotic rate of BMMSCs (*P* > 0.05, Figures 3(a)–3(c)). Apoptosis was further confirmed by Western-blotting assay, which showed a significant increase of Bax expression and a decrease of Bcl2 expression and the ratio of Bcl2/Bax (*P* < 0.01) as the cells were exposed to 1 and 10 *μ*M Ang II (Figures 3(d)–3(f)).

### 3.4. Effect of Ang II on mtROS and mtDNA Levels in BMMSCs

**Figure 4:**
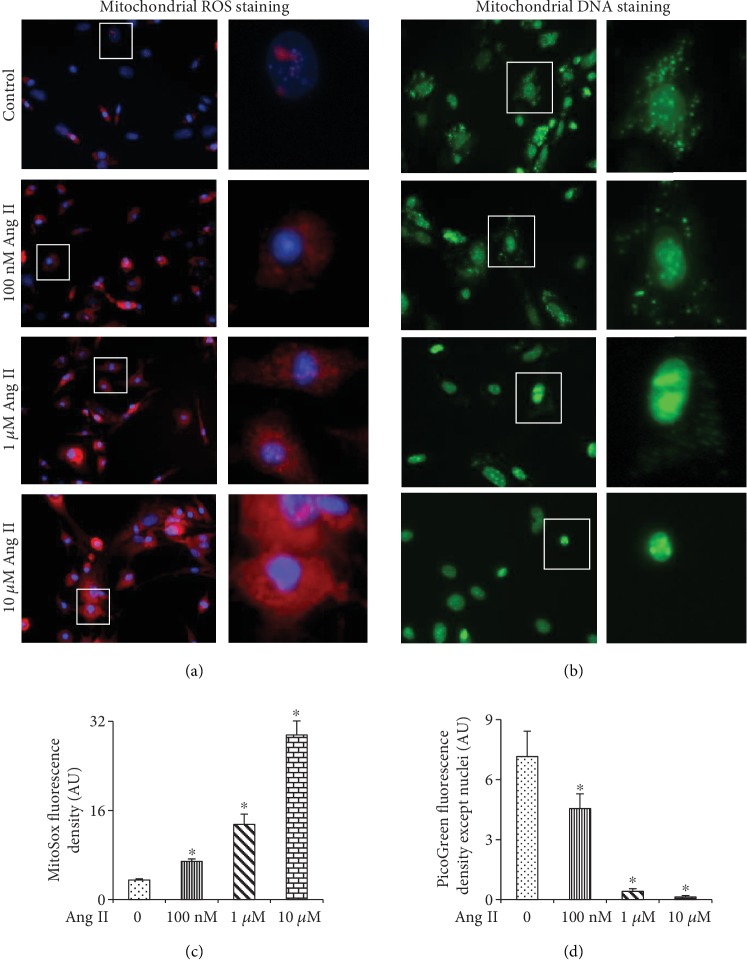
Mitochondrial ROS production and mitochondrial DNA leakage in BMMSCs after exposure to 0, 100 nM, 1 *μ*M, and 10 *μ*M Ang II for 12 hours. (a) MitoSOX™ superoxide indicator staining showing mitochondrial ROS levels in BMMSCs after exposure to different doses of Ang II. (b) PicoGreen staining showing mitochondrial DNA levels in BMMSCs after exposure to different doses of Ang II. (c) Quantification of MitoSOX fluorescence density (AU). (d) Quantification of PicoGreen fluorescence density except nuclei (AU). Bar graphs represent mean ± SD (*n* = 4 per group). ^∗^*P* < 0.01 vs. control.

As shown in Figure 4, 100 nM, 1 *μ*M, and 10 *μ*M Ang II all could induce mitochondrial ROS generation in a dose-response manner (Figure 4(a)). Moreover, 1 *μ*M and 10 *μ*M Ang II caused a marked leakage of mtDNA (less mtDNA) in BMMSCs (Figure 4(b)). Of note, mtDNA was absent in the cells with condensed or fragmented nuclei in response to 10 *μ*M Ang II (Figure 4(b), bottom panel). These data show that high concentrations of Ang II (≧1 *μ*M) can cause a marked leakage of mtDNA, which may be one of the critical reasons for apoptosis of BMMSCs induced by Ang II.

### 3.5. AT1R Signaling in Apoptosis, mtROS Generation, and mtDNA Leakage

**Figure 5:**
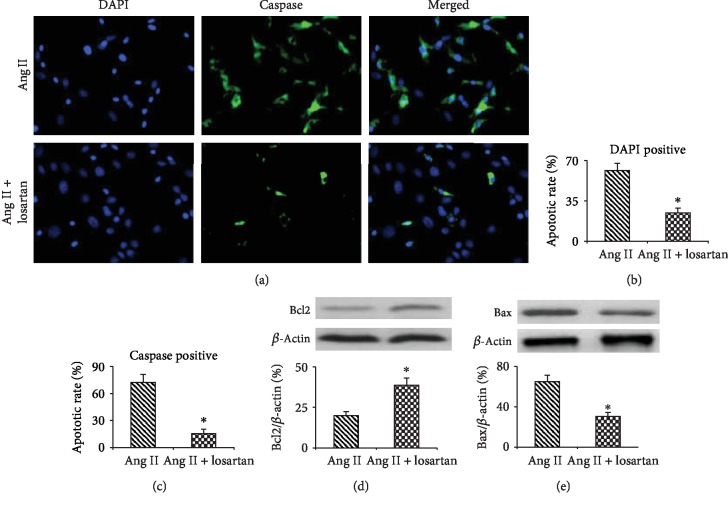
AT1R blocker losartan inhibits Ang II-induced apoptosis of BMMSCs. (a) Poly Caspase and DAPI staining showing the apoptosis of BMMSCs after pretreatment with 10 *μ*M losartan followed by exposure to 10 *μ*M Ang II. (b) Quantification of the numbers of caspase-positive cells. (c) Quantification of the numbers of the cells with condensed and/or fragmented nuclei. (d and e) Western-blotting assay showing Bcl2 and Bax expression in BMMSCs after pretreatment with losartan followed by exposure to 10 *μ*M Ang II. Bar graphs represent mean ± SD (*n* = 4 per group). ^∗^*P* < 0.05 vs. Ang II (10 *μ*M) group.

Ang II mostly exerts its action by activating its receptor AT1R. Losartan is one of the Ang II antagonists, and it achieves this by blocking AT1R. Next, we checked the role of AT1R signaling in Ang II-induced BMMSC apoptosis through the pretreatment of BMMSCs with 10 *μ*M losartan. As shown in Figure 5, pretreatment with 10 *μ*M losartan significantly inhibited Ang II-induced apoptosis of BMMSCs, downregulated Bax, and upregulated Bcl2 (*P* < 0.01). These data show that Ang II-induced apoptosis of BMMSCs is at least partially mediated by the activation of AT1R signaling.

**Figure 6:**
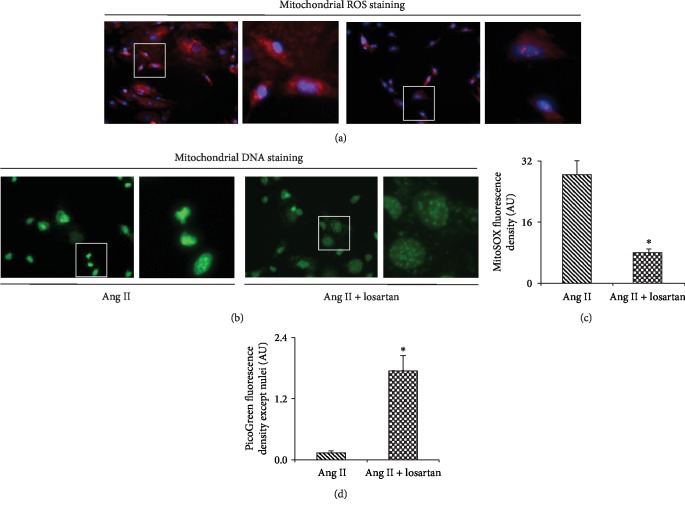
AT1R blocker losartan suppresses Ang II-induced mtROS generation and mtDNA reduction in BMMSCs. (a) MitoSOX™ superoxide indicator staining showing mitochondrial ROS levels in BMMSCs after pretreatment with 10 *μ*M losartan followed by exposure to 10 *μ*M Ang II. (b) PicoGreen staining showing mitochondrial DNA levels in BMMSCs after pretreatment with losartan followed by exposure to 10 *μ*M Ang II. (c) Quantification of MitoSOX fluorescence density (AU). (d) Quantification of PicoGreen fluorescence density except nuclei (AU). Bar graphs represent mean ± SD (*n* = 4 per group). ^∗^*P* < 0.05 vs. control.

More interestingly, Ang II-mediated mtROS production and mtDNA in BMMSCs were also markedly inhibited by treatment with losartan (Figure 6). These data indicate that the increase of mtROS and the leakage of mtDNA were caused by AT1R activation in BMMSCs.

## 4. Discussion

In this study, we showed that mtDNA leakage and mtROS production mediated by AT1R activation are responsible for the Ang II-induced apoptosis of BMMSCs. Our results showed that 1 *μ*M and 10 *μ*M Ang II could markedly increase mtROS level and cause mtDNA leakage in BMMSCs. The application of the AT1R blocker markedly inhibited mtROS production and mtDNA leakage and suppressed Ang II-induced apoptosis of BMMSCs. These findings suggest that the common doses of Ang II for the induction of cell differentiation can induce mtROS production and mtDNA reduction and subsequently cause the apoptosis of BMMSCs.

BMMSCs have been widely used as a major source of cells in tissue regenerative medicine. Ang II, a peptide hormone, is commonly used to stimulate the differentiation of MSCs to somatic cells such as cardiomyocytes, adipocytes, and smooth muscle-like cells [12, 29, 30]. However, Ang II is also a potent inducer for cell apoptosis. Previous studies have shown that Ang II can cause the apoptosis of endothelial cells, cardiomyocytes, fibroblasts, smooth muscle cells, and kidney tubular cells [8, 12, 22, 31, 32]. In this study, we found that low concentrations of Ang II (1~100 nM) promoted the proliferation of BMMSCs; however, high concentrations of Ang II (≧1 *μ*M) induced the apoptosis of BMMSCs after 12 hours of exposure. The effects of Ang II on the proliferation and apoptosis of BMMSCs in this study are similar with previous studies on other cell lines [33, 34].

A previous study had shown that Ang II could increase mtROS through the activation of Nox2 [35]. In this study, we also observed that 100 nM~10 *μ*M Ang II significantly increased the level of mtROS in BMMSCs and caused a marked leakage of mtDNA. It has been reported that mtDNA leakage triggers mitochondrial dysfunction and rapidly initiates cell apoptosis independent of mutations [36, 37]. Xie et al. reported that high glucose could cause mtROS increase and mtDNA reduction in retinal vascular endothelial cells [36]. Following mtDNA reduction, the apoptosis of endothelial cells were markedly enhanced [36]. This study suggests that the increase of mtROS results in endothelial cell apoptosis through triggering mtDNA leakage. Ricci et al. reported that mtDNA leakage could also reversely enhance mtROS generation in the cultured neonatal rat cardiomyocytes following treatment with Ang II [37]. The above studies indicate a positive feedback loop between mtROS production and mtDNA leakage, which would accelerate cell apoptosis. Ricci et al. observed that Ang II even at 1 nM could cause mtDNA leakage and apoptosis of neonatal rat cardiomyocytes [37]. However, in this study, we found that only high concentrations of Ang II (≧1 *μ*M) dramatically increased mtDNA leakage and apoptosis of BMMSCs; low concentrations of Ang II (<100 nM) did not affect the apoptosis of BMMSCs but, on the contrary, promoted their proliferation. The discrepancy between our study and Ricci's study may result from the different cell types and animal species. In addition, multiple factors may contribute to mtDNA leakage and the diminished mtDNA level including oxidative damage, which requires further studies.

Furthermore, we also found that AT1R was positively expressed in BMMSCs, and the application of the AT1R blocker markedly inhibited Ang II-induced mtROS generation, mtDNA leakage, and apoptosis in BMMSCs. This shows that Ang II-induced mtDNA reduction and BMMSC apoptosis are associated with the activation of AT1R signaling.

In summary, the present study demonstrates that higher concentrations of Ang II cause the apoptosis of BMMSCs via the activation of AT1R signaling, which is associated with mtROS production and mtDNA leakage. These findings suggest that the usually used doses of Ang II for the induction of cell differentiation have the potential to stimulate the apoptosis of BMMSCs.

## Data Availability

The data used to support the findings of this study are available from the corresponding author upon request.
